# Surgical Excision of a Traumatic Fibroma Associated With Orthodontic Temporary Anchorage Devices: A Case Report

**DOI:** 10.7759/cureus.71958

**Published:** 2024-10-20

**Authors:** Pooja Palwankar, Lakshmi Chandiran, Ruchi Pandey

**Affiliations:** 1 Periodontology, Manav Rachna Dental College, School of Dental Sciences, Manav Rachna International Institute of Research and Studies (MRIIRS), Faridabad, IND

**Keywords:** benign tumors, diode lasers, fibroma, overgrowth, tads

## Abstract

Oral fibromas typically appear as smooth bumps that match the color of the surrounding oral tissues and usually do not cause additional symptoms. They are benign tumors of fibrous tissue origin that develops as a result of continuous irritation or damage. Fibroma is the most common benign soft tissue tumor in the mouth. The primary cause is often persistent irritation from orthodontic appliances, restorative work, prosthetics, or other dental devices, which can be worsened by plaque or calculus accumulation. This case study describes a traumatic fibroma caused by the molar tubes and orthodontic temporary anchorage devices (TADs). Excision surgery was performed bilaterally using soft tissue diode laser. For the best treatment outcome, a multidisciplinary approach is always crucial in dentistry.

## Introduction

A traumatic fibroma is a common benign lesion of the oral mucosa [1]. Fibromas can occur on the skin, organs, and tissues. They can be classified as follows: plantar fibroma, non-ossifying fibroma, angiofibroma, dermatofibroma, uterine fibroid, and oral fibroma. Barker and Lucas were the first to describe the histological sections of fibroma. The differential diagnosis for these lesions may include irritational fibroma, peripheral giant cell fibroma, giant cell fibroma, fibrous nodule, focal intraoral fibrous hyperplasia, traumatic fibroma, and gingival fibromatosis. Histologically, they are mesenchymal in origin. They can be caused by chronic irritation in the area. The most common sites are the buccal mucosa, especially on the occlusion line. These lesions can be multiple and can originate from the connective tissue of the periodontium. Potential traumatic causes include overhanging margins, calculi, impacted foreign substances, restorations, persistent biting, acute bone spicules, impinging appliance borders, extended flanges of complete dentures, and other iatrogenic causes. Rather than being a genuine tumor, fibroma is known as a reactive benign growth of fibroblastic origin [2].

Fibrous polyps were initially defined in 1846 and refer to the broad soft tissue reaction to strain from teeth or dental prosthesis [3]. Many terms, including fibrous hyperplasia, focal fibrous hyperplasia, traumatic fibromatosis, localized fibrous hyperplasia, and fibrous epithelial polyp, have been used to describe non-neoplastic fibrous lesions of the oral mucosa [4]. The tongue, gingiva, and buccal mucosa are the regions most frequently involved. It typically manifests as a gradual, painless growth that happens over months to years [5]. Lesions may appear to have a broad base; the color may resemble the oral mucosa or could be lighter than the surrounding normal tissue. Rubbing against the lesion during mastication could lead to traumatic ulceration. Hyperkeratosis frequently causes the surface to appear white and could bleed on provocation, and the appearance could be sessile or pedunculated. The potential for fibroma development is limited to a radius of 5-10 mm [6]. The treatment involves excising the complete lesion, and the cause must be eradicated [7]. The rate of reversion and malignancy is low and can be attributed to the repetitive trauma of the site.

## Case presentation

An 18-year-old female patient was referred to the Department of Periodontology for abnormal soft tissue growth in her right and left upper front teeth region in the oral cavity. Intraoral examination and discussion with the patient revealed that the growth had increased slowly over eight months, without causing concern initially.

**Figure 1:**
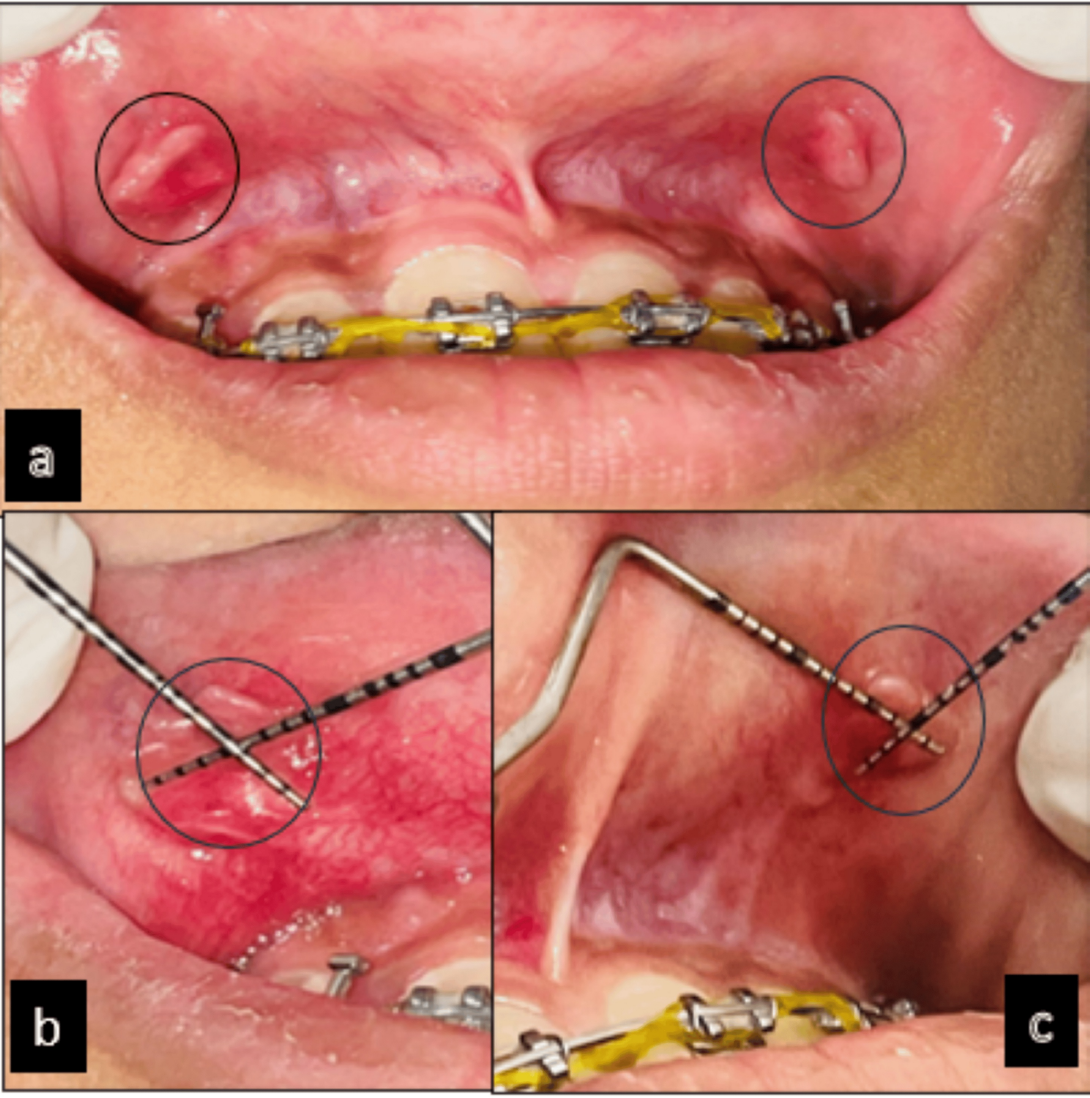
Preoperative view. (a) Lesion on the right and left maxillary labial mucosa. (b) Lesion measuring 7x5x4 mm on the right maxillary labial mucosa. (c) Lesion measuring 6x3x6 mm on the left maxillary labial mucosa.

The pain intensified when the mucosa was stretched during smiling and mastication. The growth was located in the labial mucosa bilaterally. A detailed case history disclosed that the patient did not have significant medical or family history. The dental history revealed that the patient had been undergoing orthodontic treatment for the past two years. On examining intraorally, a pedunculated, well-defined bilateral growth was observed over the left and right labial mucosa on the facial aspect opposite the maxillary right and left lateral incisor and canine, with the growth extending from teeth 12 to 13 and 22 to 23 bilaterally (Figure 1a). The lesion on the right maxillary mucosa opposite 12 and 13 measured approximately 7x5x4 mm (Figure 1b). The lesion on the left maxillary mucosa opposite 22 and 23 measured approximately 6x3x6 mm (Figure 1c). The lesions were measured using the University of North Carolina (UNC-15) probe (HuFriedy Group, Chicago, IL, USA). 

On palpating the growth, it was painless and firm in consistency. Temporary anchorage devices (TADs) were placed in relation to 12, 13 and 22, 23, and buccal tubes were placed on 23 and 13, which might be irritating the labial vestibule. The cervical lymph nodes were non-palpable. An orthopantomogram (OPG) was advised, which showed no abnormality. The oral cavity was disinfected using 0.2% chlorhexidine gluconate (Hexidine, ICPA Health Products Ltd, India). A local anesthesia sensitivity test was conducted, after which topical anesthesia, 2% lidocaine hydrochloride gel (2% LOX, Neon Laboratories Ltd, Mumbai, India), was applied at the injection site. This was followed by the administration of local anesthesia containing 2% lignocaine hydrochloride with adrenaline 1:80000 (Lignox 2% A, Indoco Remedies Ltd, New Delhi, India).

**Figure 2:**
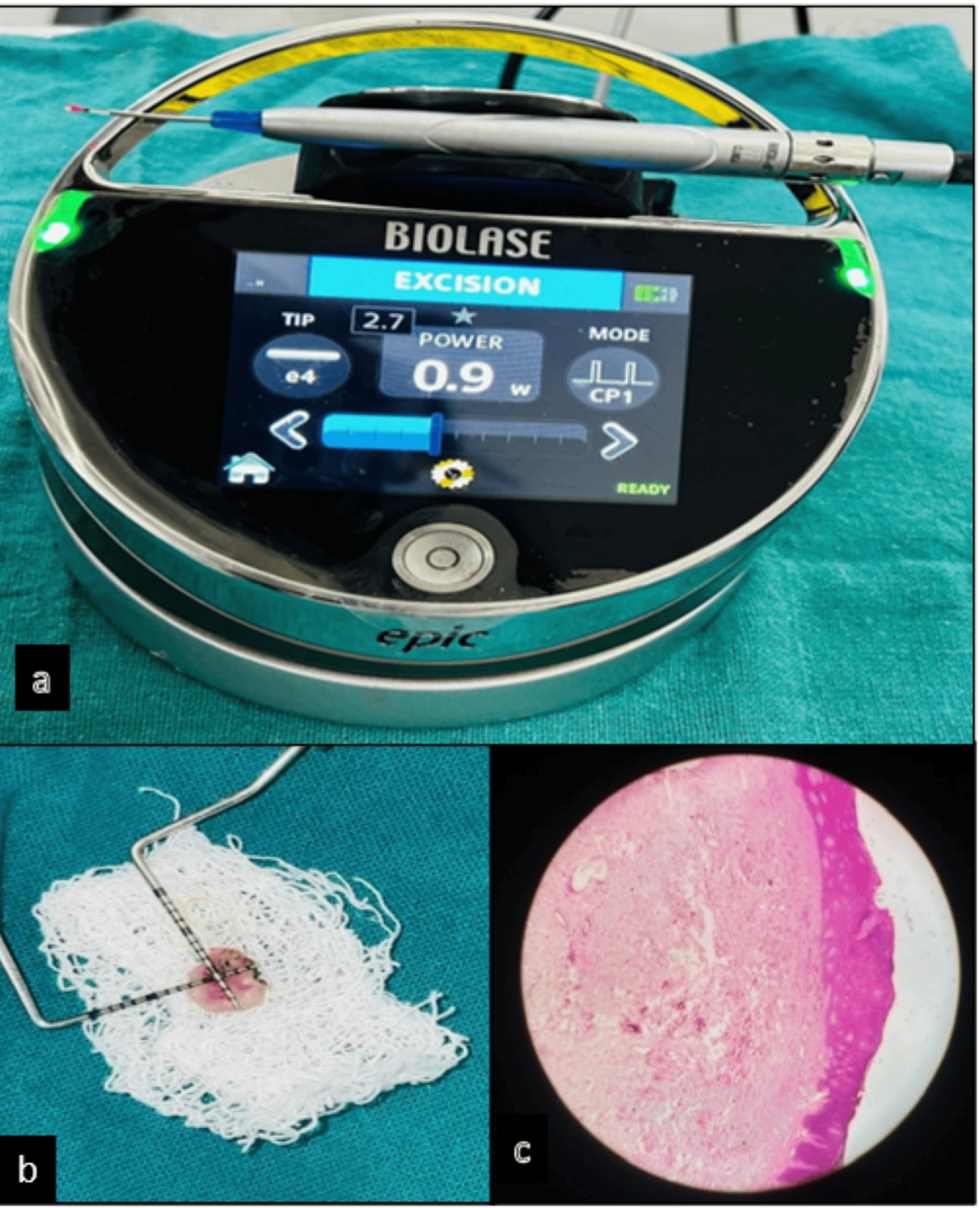
Surgical excision. (a) Diode laser settings. (b) Excised lesion. (c) Histological slide with stratified squamous epithelium and fibroblasts.

The complete intraoral excision was performed bilaterally using the LASER (light amplification by stimulated emission of radiation) with a wavelength of 940 nm (Biolase Epic X-Diode Laser, BIOLASE Inc, Foothill Ranch, CA, USA), at a power of 0.9 watts, in e4 and CP1 mode (Figure 2a). The lesions were excised (Figure 2b). The lesions were fixed in 10% neutral-buffered formalin (NBF) and sent for histopathological analysis (Figure 2c). 

**Figure 3:**
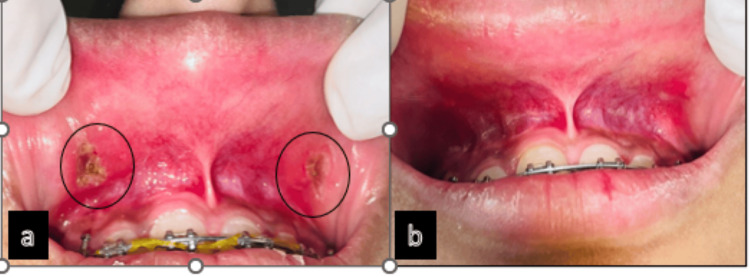
Postoperative view. (a) Immediate postoperative view. (b) One-month follow-up.

The immediate postoperative picture was recorded (Figure 3a). The patient was recalled for postoperative evaluation after one month (Figure 3b). The lesions had healed without any postoperative complications. The immediate postoperative and follow-up periods were uneventful.

## Discussion

Localized fibrous tissue overgrowths are 1-2% prevalent in the oral mucosa. The etiology associated with traumatic fibroma is mainly irritation from various factors. According to Barker and Lucas, irritational fibromas display a pattern of collagen organization that depends on the location and degree of irritation. Two categories of patterns exist: radiating and circular. It is postulated that higher levels of stress cause the former to exist in inherently static locations (like the palate), while lower levels of trauma cause the latter to occur in naturally flexible locations (like the buccal mucosa) [2]. Similar lesions can develop due to irritation caused by dental plaque and local tissue factors, which include pyogenic granuloma, peripheral giant cell granuloma, and peripheral ossifying fibroma [7]. Each lesion has a comparable clinical and histological appearance [8]. Treatment of traumatic fibroma involves complete excision of the growth to reduce the likelihood of the recurrence, followed by scaling and curettage of the neighboring area and removal of irritants. The principal rule is to eliminate known irritants.

In this case report, the lesions occurred due to continuous irritation from the TADs and the molar tube. Following the surgical removal of the lesions, orthodontic treatment for bite correction was intended to prevent additional stress at the lesion site due to occlusion; therefore, the molar tubes and TADs were removed.

Traumatic lesions frequently occur in the oral cavity, with a high predilection for females, and have a prevalence rate of 1-2% [9]. The predominant site is the occlusal line of the buccal mucosa, but they can appear in other sites as well, with a size of approximately 1.5 cm [10]. If left untreated, they increase in size. A study by de Santana Santos et al. found that 15% of 1,290 soft tissue oral lesions were traumatic fibromas. In females, hormones play a crucial role in increasing collagen synthesis [4].

The use of diode laser for excision showed drastic improvement in the patient’s condition and has become the choice of treatment [11]. The recurrence rate of the lesion remains low if the irritant is identified and eliminated with enucleation of the lesion and regular follow-ups.

## Conclusions

A thorough case history, oral examination, and correct diagnosis are key to successful treatment. It is also crucial to choose an effective treatment modality that minimizes postoperative complications and is beneficial in preventing recurrence. Iatrogenic causes should be reduced through careful planning and regular follow-up of treated cases. Therefore, a comprehensive multidisciplinary oral healthcare approach is essential for managing surgical lesions.
